# Editorial: Changing Perspectives on Landscape Perception: Seeking Common Ground Between the Psychological Sciences and the Humanities

**DOI:** 10.3389/fpsyg.2020.00159

**Published:** 2020-02-11

**Authors:** Laura Menatti, Harry Heft

**Affiliations:** ^1^Ecole Nationale Supérieure d'Architecture et de Paysage de Bordeaux, Talence, France; ^2^UMR5319 Passages UMR, Pessac, France; ^3^Department of Psychology, Denison University, Granville, OH, United States

**Keywords:** landscape, perception, ecological psychology, aesthetics, philosophy, affordances, visual perception, geography

The history of the concept of landscape is intimately tied to interrelated developments in the visual arts, photography, aesthetics, and the scientific study of visual perception. For a long time, landscape has been typically considered as a visually pleasing view of an expanse in a natural or urban setting (e.g., the so-called *veduta;* Menatti, 2017). It is usually taken to be a beautiful view afforded to a spectator, someone who looks on it from a fixed position, and as such, it is commonly conceptualized in picture-like, or image-based terms (Heft, 2010).

Nevertheless, studies both in natural sciences and humanities have questioned a narrow image-based approach to thinking about landscape. In psychology, philosophy, and cognitive science, for one thing, approaches to visual perception over the past half century have begun to recognize the ways in which actions of the body participate in visual experience, and they have challenged the notion of detached, stationary spectator of the environment. Along with phenomenology (Husserl, 1913; Merleau-Ponty, 1962) the idea of perception has evolved to imply the role of the whole body and not just the eye, the optic tract, and projection areas in the brain. Gibson's ecological approach to perception was influenced by phenomenological writings, especially by the Gestalt psychologist (Heft, 2001). The enactive approach in cognitive science (Varela et al., 1991) has been shaped by how phenomenology as conceived the perceiver as being embedded (live from inside) in the *lifeworld*.

In aesthetics, geography and landscape theory an analysis has been conducted to interrelate landscape painting/representation to landscape perception (Gombrich, 1966; Berque, 1995, 2013; Carlson, 2000; Cosgrove, 2007; Briffaud, 2014). The contemplative and aesthetic value of landscape (developed through Romanticism and Idealism in philosophy) which has been for many year the main approach to discuss landscape, has been widened to include political analysis, such as through the lens of the history of commons (Olwig, 1996, 2002). Finally, thanks to the formulation of the related legal documents (such the European Landscape Convention, 2000), there is recognition that the concept of landscape goes beyond considerations of visual perception to include socio-political and cultural factors that are tied to traditions of community life inseparable from ecological and resource conditions.

The complexity of the analysis of the concept of landscape developed in philosophy, geography, and psychology has led to an emphasis on the experience of the environment from the standpoint of the perceiver-as-agent developing and living within a particular socio-historical context. As participants in local contexts, individuals are exposed to narratives, discourses, and histories pertaining to landscape.

On these grounds, it is necessary to go beyond solely image-based considerations to recognize the multitude of factors that contribute to an individual's experience of landscape. Such a perspective calls for an interdisciplinary approach. With this stance in mind, we attempted to widen the perspective on landscape perception through the Frontiers Topic “*Changing Perspectives on Landscape Perception: Seeking Common Ground Between the Psychological Sciences and the Humanities*.” This initial attempt in doing so led us to solicit contributions by geographers, philosophers, environmental psychologists, architects, and ecological psychologists around the important question “how do we perceive landscape?” The results are six papers collected here.

Various approaches emerged from our Frontiers Topic. A narrative and discourse-based approach characterizes the paper by Aliste et al.. Coming from the geographical and architectural school of Universidad de Chile, the authors describe a landscape in which different political practices (not least the Pinochet dictatorship) have molded the territories of Southern Chile. By following also a constructivist and philosophical approach, the authors describe the discourses that have influenced and determined the different landscapes' practices, thus conveying a representationalist and political approach to perception.

A non-representationalist approach to landscape perception emerges from the papers by Heras-Escribano and De Piñedo García and the paper by Rietveld and Rietveld. A non-representationalist approach to landscape relies on ecological psychology as fundamental tool in understanding visual perception. This approach was developed by James Gibson (Gibson, 1979), and it has the effect of naturalizing landscape perception (see Menatti and Casado da Rocha, 2016), embedding it in biological and adaptive concepts such as affordances and niche construction.

In the paper by Heras-Escribano and Pinedo-García niche construction theory (NCT) is used to overcome the nature–culture dualism of the relationship between human being and the environment. Stuck in the opposition between objective qualities of the environment or subjective perception of the spectator, landscape has been often reduced to an artistic object to contemplate. In the mentioned paper, NCT finds a solution to the previous dualism and considers the actual activities that we engage as perceivers in the landscape. Perception is thus described through the lens of affordances and resulting in a *processual landscape* (a term introduced by Menatti and Casado da Rocha, 2016). Even more the concept of a niche is further detailed in NCT as implies human (both cultural and biological) and environmental elements. In determining and creating a landscape—which is an ever-evolving part of the human environment—both socio-cultural and biological-natural based elements are interrelated and co-determined in the course of evolution.

Furthermore, ecological psychology has also been employed from an architectural point of view, for what concerns the conservation and the management of heritage in a specific landscape. It is the case of the paper by Rietveld and Rietveld, in which the conservation approach to heritage has been questioned through the lens of affordances and embodied perception to promote a unique and original “hardcore heritage” intervention on a bunker on the Dutch coast.

A third paper which takes up architectural matters from the perspective of ecological psychology is Withagen and Caljouw analysis of the influential abstract forms applied to playscapes that were developed by the Dutch architect Aldo van Eyck in post-war Amsterdam in order to foster creativity in children. Their paper approaches these designs from the perspective of affordances.

This Frontiers Topic was also illuminated by recent research in environmental psychology which has focused on the possible beneficial and the restorative effects of landscape. In this regard, the paper by San Juan et al. focuses on the restorative potential of urban settings, namely urban squares. The paper discusses the restorative role of urban and natural landscapes, by demonstrating that cities can be potentially restorative and that the concept of landscape can be widened to include urban design characteristics.

The paper by Hägerhäll et al. analyzes cross-cultural landscape preferences in indigenous and non-indigenous populations. It focuses on the topic of the universal consensus in landscape preferences, based on theories such as Savannah Theory, the Prospect Refuge Theory, Biophilia etc. The results demonstrate a difference between Western and Non-Western samples: the former prefer moderate opened landscapes, the latter one with high vegetation density. The reasons for preference may depend on differences in settings, schooling, place attachment, and also linguistic samples in defining landscape. Claims of innate preferences, driven for instance by evolution, are not fully supported and the cultural elements in determining landscape preferences need to be further explored.

In conclusion, the heterogeneity of these papers reflect the interdisciplinary character of the concept of landscape itself. These papers are a contribution to a much needed interdisciplinary discussion and analysis of this important concept.

## Author Contributions

All authors listed have made a substantial, direct and intellectual contribution to the work, and approved it for publication.

### Conflict of Interest

The authors declare that the research was conducted in the absence of any commercial or financial relationships that could be construed as a potential conflict of interest.
